# Arterial Resection for Pancreatic Cancer: Feasibility and Current Standing in a High-Volume Center

**DOI:** 10.1097/AS9.0000000000000302

**Published:** 2023-06-28

**Authors:** Lei Ren, Carsten Jäger, Stephan Schorn, Ilaria Pergolini, Rüdiger Göß, Okan Safak, Maximilian Kießler, Marc E. Martignoni, Alexander R. Novotny, Helmut Friess, Güralp O. Ceyhan, Ihsan Ekin Demir

**Affiliations:** From the * Department of Surgery, Klinikum rechts der Isar, Technical University of Munich, School of Medicine, Munich, Germany; † Department of General Surgery (Gastrointestinal Surgery), The Affiliated Hospital of Southwest Medical University, Luzhou, Sichuan, China; ‡ German Cancer Consortium (DKTK), Partner Site Munich, Munich, Germany; § CRC 1321 Modelling and Targeting Pancreatic Cancer, Munich, Germany; ‖ Department of General Surgery, HPB-Unit, School of Medicine, Acibadem Mehmet Ali Aydinlar University, Istanbul, Turkey; ¶ Else Kröner Clinician Scientist Professor for Translational Pancreatic Surgery, Munich, Germany.

**Keywords:** arterial resection, pancreatic cancer, R0 resection, venous resection

## Abstract

**Background:**

Arterial resection (AR) during pancreatectomy for curative R0 resection of pancreatic ductal adenocarcinoma (PDAC) remains a controversial procedure with high morbidity.

**Objective:**

To investigate the feasibility and oncological outcomes of pancreatectomy combined with AR at a high-volume center for pancreatic surgery.

**Methods:**

We retrospectively analyzed our experience in PDAC patients, who underwent pancreatic resection with AR and/or venous resection (VR) between 2007 and 2021.

**Results:**

In total 259 PDAC patients with borderline resectable (n = 138) or locally advanced (n = 121) PDAC underwent vascular resection during tumor resection. From these, 23 patients had AR (n = 4 due to intraoperative injury, n = 19 due to suspected arterial infiltration). However, 12 out of 23 patients (52.2%) underwent simultaneous VR including 1 case with intraoperative arterial injury. In comparison, 11 patients (47.8%) underwent AR only including 3 intraoperative arterial injury patients. Although the operation time and bleeding rate of patients with AR were respectively longer and higher than in VR, no significant difference was detected in postoperative complications between VR and AR (*P* = 0.11). The final histopathological findings of PDAC patients were similar, including M stage, regional lymph node metastases, and R0 margin resection. The mortality of the entire cohort was 6.2% (16/259), with a tendency to increase mortality in the AR cohort, yet without statistical significance (VR: 5% *vs* AR: 21.1%; *P* = 0.05). Although 19 (82.6%) patients had PDAC in the final histopathology, only 6 were confirmed to have infiltrated arteria. The microscopic distribution of PDAC in these infiltrated arterial walls on hematoxylin-eosin staining was classified into 3 patterns. Strikingly, the perivascular nerves frequently exhibited perineural invasion.

**Conclusions:**

AR can be performed in high-volume centers for pancreatic surgery with an acceptable morbidity, which is comparable to that of VR. However, the likelihood of arterial infiltration seems to be rather overestimated, and as such, AR might be avoidable or replaced by less invasive techniques such as divestment during PDAC surgery.

## Abstract 2

Arterial resection (AR) during pancreatectomy for curative R0 resection of pancreatic ductal adenocarcinoma (PDAC) remains a controversial procedure. Here, we report the outcome of patients who underwent AR over the past 14 years in our institution and propose a novel classification for arterial wall invasion in PDAC, which may guide future decision-making during AR in pancreatic surgery.

## INTRODUCTION

Surgical resection is the cardinal effective therapy for patients with resectable pancreatic ductal adenocarcinoma (PDAC) and without major infiltration of important visceral arteries.^1,2^ Although Fortner et al^3^ first proposed an extended surgical approach to pancreatectomy that combines arterial resection (AR) and venous resection (VR) in 1973, the remarkably morbid outcomes of pancreatectomy with vascular resection have still remained the main factor restricting the development of pancreatic surgery since then. Owing to the implementation of thorough preoperative workup, improved perioperative care, new multimodal treatment regimens, and improved technical approaches during surgery,^4–7^ pancreatic surgery combined with VR in high-volume centers (HVCs) has remarkably improved the oncological outcomes. However, pancreatectomy combined with AR remains controversial.^8^ Nearly 50% of patients with PDAC are diagnosed at an advanced stage,^9^ which is defined as tumor encasement of the celiac artery (CA), common hepatic artery, or superior mesenteric artery. R0 resection is an independent prognostic factor for overall survival in PDAC,^10^ and is the only chance for long-term survival for patients with locally advanced (LA) PDAC.^6,11^ As such, pancreatic surgeons are continuously striving for new strategies to increase the chances of R0 resection.^8,12,13^ Attaining an R0 status frequently requires vascular resection,^10,14^ including, in some cases, also an AR. However, arterial infiltration has been classically regarded as a contraindication to surgery, which can be ascribed to the high morbidity and mortality associated with AR and/or reconstruction.

Ostensibly, PDAC, especially pancreatic head cancer, can frequently and directly infiltrate the superior mesenteric vein and portal vein (PV) by local tumor extension. Intrinsically, PDAC is a malignancy associated with venous thrombosis, which is linked with 17%−36% morbidity.^15–20^ The frequent occurrence of venous thrombosis in PDAC patients is ascribed to early and excessive activation of platelets and procoagulant factors.^21,22^ In patients receiving AR and/or arterial reconstruction, the intraoperative blood loss aggressively stimulates the stress responses and feedback mechanisms in the body, thereby further promoting prothrombin activation and platelet activation to compensate for blood loss.^21,22^ The hemodynamic variations provide a condition for platelet aggregation, which is caused by the hypercoagulable state of blood in advanced cancers,^23^ temporary blood flow blockage during microvascular anastomosis, or the blood eddy currents in vascular stumps. Moreover, anastomotic sutures will create attachment points for platelet aggregation. Notably, visceral thrombosis, predominantly portal vein thrombosis,^22,24^ usually occurs in PDAC and has been identified as a prognostic factor for short-term survival.^25^ Moreover, the expansion of portal vein thrombosis into the liver can cause intrahepatic thrombosis, leading to impaired liver function and even liver failure.^22,24,26–28^

The present study assessed the perioperative morbidity and mortality, postoperative complications, and the long-term oncologic outcome in borderline resectable (BR)/LA PDAC patients who received AR and/or VR during pancreatectomy in an HVC for pancreatic surgery.

## METHODS

### Study Approval

The retrospective analysis of the perioperative and oncological outcome of our resected PDAC patients was approved by the Ethics Committee of the Technical University of Munich (nr. 2022-407-S-NP).

### Patient Cohort

We retrospectively reviewed our electronic database of 138 BR and 121 LA PDAC patients who underwent pancreatic resection at Klinikum rechts der Isar, Technical University of Munich, Germany, between 2007 and 2021. The following clinicopathologic data were collected to analyze prospectively: baseline characteristics (age, sex), surgical procedures [pancreatoduodenectomy (PD), proximal pancreatectomy, distal pancreatectomy (DP), or total pancreatectomy (TP)], operative time, postoperative complications [Clavien–Dindo] and intraoperative bleeding classification, mortality, histopathological findings [TNM status, tumor grade, resection margin status (R0/R1/Rx)^29]^, neoadjuvant/adjuvant treatment, survival, and vascular (venous and/or arterial) resection (including the cause and type). All the relevant histopathological findings were obtained from the pathology reports.

### Preoperative Diagnostic Evaluation and Inclusion Criteria

Preoperative baseline radiology staging for all patients was performed by using triple-phase thorax/abdomen/pelvis computed tomography (CT), which led to the categorization of the patients as BR or LA PDAC according to the NCCT guidelines.^30^ The type of surgical procedure was dependent on the tumor localization and varied between PD, DP, or TP. For analysis of AR feasibility, all patients who received AR during pancreatectomy were included. The decision for surgery was met after a discussion in our multidisciplinary tumor board.

### Surgical Procedures

**FIGURE 1.**
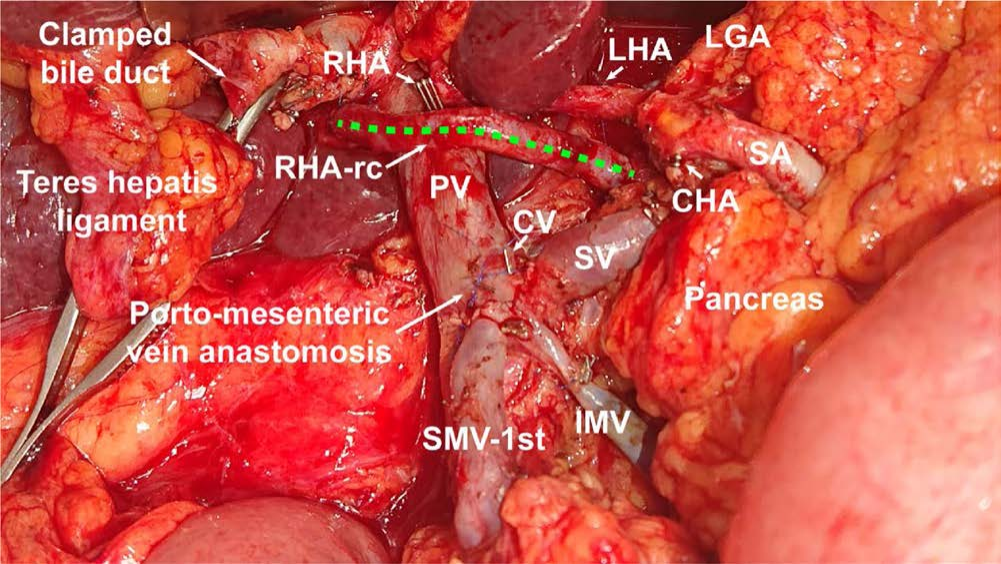
Ovarian vein graft in the course of the reconstructed right hepatic artery in pancreatic head cancer during pancreatoduodenectomy combined with hepatic artery resection. CV indicates coronary vein; IMV, inferior mesenteric vein; LGA, left gastric artery; LHA, left hepatic artery; PV, portal vein; RHA, right hepatic artery; RHA-rc, ovarian vein graft in the course of the reconstructed right hepatic artery; SA, splenic artery; SMV-1st, 1st order branch of superior mesenteric vein; SV, splenic vein.

We employed the artery-first approach,^31^ and intraoperative frozen sections were utilized in all cases for suspected arterial infiltration and for the parenchymal resection margin. The vascular resection involved one or more of the following vessels: superior mesenteric vein, portal vein, hepatic artery (HA), celiac trunk, superior mesenteric artery, and spleen artery. The venous and arterial reconstruction was performed as (1) end-to-end anastomoses, or (2) by means of autologous or synthetic grafts (Fig. 1). All surgical procedures were performed by 1 of 4 experienced pancreatic surgeons of our institution.

### Outcome Assessment

The postoperative complications were graded according to the Clavien–Dindo classification and the International Study Group of Pancreatic Surgery classification. The discharge norm was based on the patients taking a normal solid diet for at least 3 days and having no discomfort after the extraction of the peritoneal drainages (2 easy-flow drains placed around the pancreatic and biliary anastomoses). The arterial and/or vein thrombosis was routinely ruled out before discharge by ultrasound and/or CT angiography. Median survival was calculated from the date of surgery, and the serum tumor marker measurement and the radiologic follow-up (CT or magnetic resonance imaging) were performed every 3 months during year 1 and year 2, then every 6 months during years 3–5, and annually after 5 years.

### Hematoxylin-Eosin Staining

Although 4% paraformaldehyde-fixed, paraffin-embedded resection specimens were used for the histopathological The sections were deparaffinized 3 times with Roticlear and rehydrated in different concentrations of ethanol. After staining of the sections in hematoxylin and eosin, we dehydrated them with ethanol and Roticlear.

### Statistical Analysis

Descriptive statistics were performed using the case number/percentage for discrete variables and the median/mean for continuous variables. For the analysis of association, we used *χ*^2^ or student’s *t* test or Mann-Whitney-U-test as appropriate. All statistical analyses were performed by using the *P* value <0.05 as statistical significance.

## RESULTS

### Patient Characteristics and Perioperative Data

A total of 259 BR/LA PDAC patients (BR: n = 138; LA: n = 121) with vascular resection from 2007 to 2021 were enrolled in our study. From these, 23 patients had AR (n = 4 due to intraoperative injury, n = 19 due to suspected arterial infiltration). However, 12 out of 23 patients (52.2%) underwent simultaneous VR including 1 case with intraoperative arterial injury. In comparison, 11 patients (47.8%) underwent AR only including 3 intraoperative arterial injury patients.

There were 7 (36.8%) patients with AR and 49 (20.4%) with VR ultimately receiving surgical resection after neoadjuvant chemotherapy, which included 5 patients with only Folfirinox and 2 patients with Folfirinox and Gemcitabine plus Abraxane in the AR cohort. The detailed patient baseline characteristics are summarized in Table 1. These baseline clinical characteristics of patients who underwent VR and/or AR during pancreatic surgery were comparable, with no prominent variation. The surgical resection types included 162 pancreatoduodenectomies/PD (Whipple), 66 TP, and 31 DP. Among the PDAC patients who underwent VR alone, 155 received PD (64.6%), 60 had TP (25%), and 25 achieved DP (10.4%). In patients undergoing the AR alone (n = 19), 7 underwent PD (36.8%), 6 had TP (31.6%), and 6 had DP (31.6%). The median operation time of PDAC patients with AR (537 mins) was longer than that of patients with VR (429 mins) (*P* = 0.001).

**TABLE 1.** Clinicopathological Data

Variable	Vein Resection (n = 240)		Arterial Resection (n = 19)		*P*
Sex					0.11
Male	128	(53.3%)	9	(47.4%)	
Female	112	(46.7%)	10	(52.6%)	
Age, mean (STD)	67.3 (10.4)		63.7 (11.0)		0.24
Neoadjuvant CTx	49	(20.4%)	7	(36.8%)	0.1
Operation					0.09
ppWhipple/Whipple	155	(64.6%)	7	(36.8%)	
TP	60	(25%)	6	(31.6%)	
DP	25	(10.4%)	6	(31.6%)	
Operative time (min), mean (STD)	429 (111)		537 (131)		**0.001**

CTx indicates chemotherapy; DP, distal pancreatectomy; TP, total pancreatectomy.

### Perioperative Outcomes

In the AR cohort, 8 out of 19 patients (42.1%) underwent AR only, and 11 patients (57.9%) underwent simultaneous VR. No difference was present with regard to tumor grade or M stage among PDAC patients with VR *versus* AR only *versus* AR and VR [M0: 225 (93.8%) *vs* 7 (87.5%) *vs* 9 (81.8%); M1: 15 (6.3%) *vs* 1 (12.5%) *vs* 2 (18.2%)]. Regional lymph node metastases were identified in 180 patients with (75%) VR only,4 (50%) with AR only, and 9 (81.8%) with AR and VR. The distribution of T1/T2 and T3/T4 were also comparable between patients with VR AR only, AR and VR (57.1% *vs* 75% *vs* 54.5%, 42.9% *vs* 25% *vs* 45.5%, separately). R0 resection was achieved in 21.6% (56/259) of all patients [including 52 (21.7%) patients with VR, 3 (37.5%) with AR only, and 1 (9.1%) with AR and VR]. We detected a tendency toward increased mortality in the AR group, yet without statistical significance (VR: 5% *vs* AR only: 9.1% *vs* AR and VR: 25%; *P* = 0.33). The patients mainly died due to postoperative hemorrhage in the AR cohort. The postoperative bleeding rate after AR [7 (36.8%), including 3 (27.3%) with AR only and 4 (33.3%) with AR and VR] was significantly higher than that after VR (7.9) (X2-test, *P* = 0.01). Surgery-related complications including pancreatic fistula, biliary fistula, and intestinal fistula showed no significance between VR and AR cohorts (Table 2).

**TABLE 2.** The Perioperative Outcomes of Arterial *Versus* Venous Resection in PDAC

Variable	Venous Resection (n = 240)		Arterial Resection Only (n = 8)		Arterial and Venous Resection (n = 11)		*P*
Pathological findings							
M-Status							0.7
M0	225	(93.8%)	7	(87.5%)	9	(81.8%)	
M1	15	(6.3%)	1	(12.5%)	2	(18.2%)	
T-stage							0.4
T1/T2	137	(57.1%)	6	(75%)	6	(54.5%)	
T3/T4	103	(42.9%)	2	(25%)	5	(45.5%)	
Nodal status							0.16
0	60	(25%)	4	(50%)	2	(18.2%)	
1	180	(75%)	4	(50%)	9	(81.8%)	
Resection margins							0.17
R0	52	(21.7%)	3	(37.5%)	1	(9.1%)	
R1	169	(70.4%)	4	(50%)	7	(63.6%)	
Rx	19	(7.9%)	1	(12.5%)	3	(27.3%)	
Grading							0.7
G1/G2	116	(48.3%)	3	(37.5%)	7	(63.6%)	
G3/G4	94	(39.2%)	5	(62.5%)	1	(9.1%)	
Not assessed	30	(12.5%)	0	0	3	(27.3%)	
Postoperative complications							0.6
Grade 0/I/II	167	(69.6%)	4	(36.4%)	5	(41.7%)	
Grade III/IV	61	(25.4%)	6	(54.5%)	4	(33.3%)	
Mortality (30-day/in-hospital)	12	(5.0%)	1	(9.1%)	3	(25%)	0.05
Bleeding B/C	19	(7.9%)	3	(27.3%)	4	(33.3%)	0.3
Pancreatic fistula B/C	25	(10,4%)	2	(25%)	2	18.2%	
Biliary fistula	8	(3.3%)	1	(4.3%)	0	0	
Intestinal fistula	0	0	0	0	0	0	

In addition to the 19 PDAC patients who underwent AR, there were 4 cases with intraoperative arterial injury, which compelled us to perform AR only in 1 patient, and simultaneously AR and VR in 3 patients. In fact, these patients suffered from a recurrence of gastric cancer infiltrating the pancreas, chronic pancreatitis, bile duct cancer, or pancreatic neuroendocrine tumor. The types of AR in our all AR were celiac trunk resection (n = 5), hepatic artery resection (n = 16), and other AR types (n = 2). Furthermore, only in 6 (1 patient underwent AR only and 5 patients underwent AR and VR) out of 19 cases with PDAC, the resected artery was microscopically infiltrated (Table 3).

**TABLE 3.** The Indications for Arterial Resection

Arterial Resection	Type		No. of Cases
Cause	Injury (non-PDAC)	(AR, only)	3
		(AR and VR)	1
	Suspicion of tumor infiltration (PDAC)		19
Type	Celiac trunk		5
	HA		16
	Other		2
Infiltrated arteria in pathology	Yes	AR only	1
		AR and VR	5
Pathological findings	Non-PDAC		4
	PDAC		19

AR indicates arterial resection; HA, hepatic artery; PDAC, pancreatic ductal adenocarcinoma; VR, venous resection.

### Long-Term and Oncologic Outcomes

Although the complication rate in the AR cohort was higher than that in the VR cohort, no significant difference was detected between VR and AR (*P* = 0.11). The mortality rate of the entire cohort was 6.2% (16/259). In the AR cohort, 4 deaths due to surgical complications were reported (3 patients within the first 30 days in the hospital, 3 of which resulted from relevant bleeding and its complications). There were 4 cases of liver complications, namely, liver abscess, hepatic encephalitis, thrombosis, and sepsis. The overall survival in AR presented likewise poor, all 4 non-PDAC-patients died due to complications respectively within 30 days, after 3, 5, and 12 months; in the 19 PDAC-AR cohort, 4 patients died due to complications, whereas 9 died after a median survival of 14.8 months, and only 2 patients had a recent survival of 48.8 and 65 months, but both of them developed tumor recurrence.

### Microscopic Distribution of PDAC Around the Resected Arteries

**FIGURE 2.**
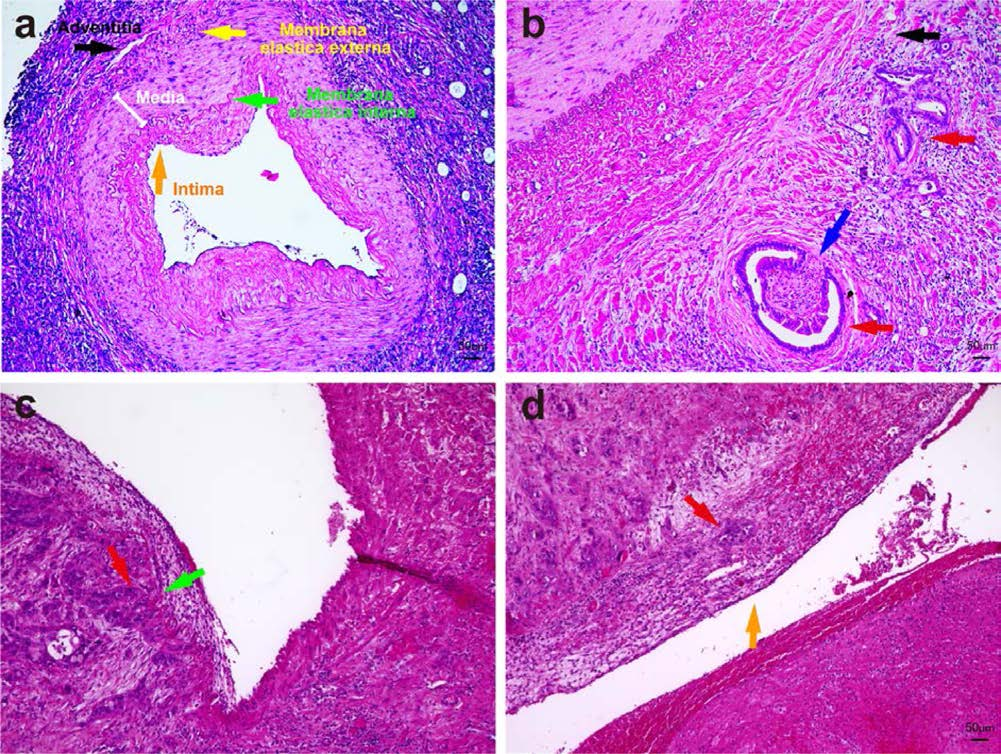
Extent of artery infiltration by pancreatic cancer cells. A, The typical micromorphology of visceral arteries. B, Cancer infiltrating the arterial adventitia. C, Cancer infiltrating the arterial media and clung to membrana elastica interna. D, Cancer infiltrating the arterial intima. blue arrow, * Nerve/ perineural invasion; red arrow, cancer cells; yellow arrow, membrana elastica external; green arrow, membrana elastica interna; orange arrow, arterial intima.

The pattern of arterial invasion can have consequences for the intraoperative surgical technical approach to the artery. Therefore, the distribution of tumor cells in the arterial wall was also analyzed based on hematoxylin-eosin-stains of tumor-invaded arteries (Fig. 2A). Three types of artery infiltration by PDAC cells were identified in our PDAC-AR cohort (Table 4), namely, (1) 1 case with cancer cells invading arterial adventitia (Fig. 2B); (2) 4 cases with cancer cells invading the arterial media and membrana elastica interna (Fig. 2C); and (3) 1 case with cancer cells invading the arterial intima (Fig. 2D). Interestingly, in all the cases of arterial infiltration, the perivascular nerves exhibited perineural invasion (Fig. 2B). Thus, in line with our previous reports concerning the particular propensity for perineural invasion in PDAC,^32–35^ peripheral nerves distributed along the visceral vessels might be among the key factors that lead to the clinically detected arterial infiltration around superior mesenteric artery, HA, and celiac trunk.^36^ Hence, in the analysis of our cohort, the majority of the infiltrated arteries exhibited a Type 2 infiltration, that is, invasion of the arterial media (Table 4).

**TABLE 4.** The Artery Infiltration Types in Our PDAC-AR Cohort

**Type**	**No. of Cases**
Cancer cells invading arterial adventitia	1
Cancer cells invading the arterial media and membrana elastica interna	4
Cancer cells invading the arterial intima	1

**FIGURE 3.**
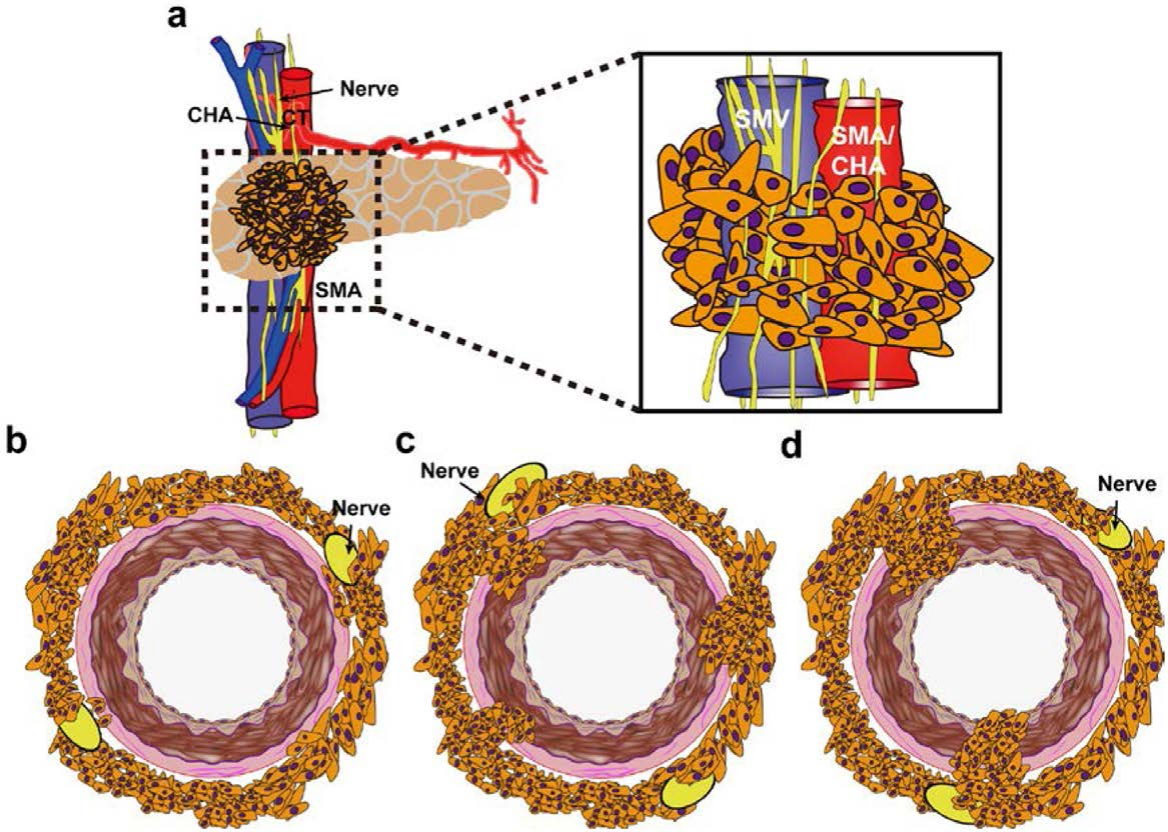
Schematic diagram of the artery invasion patterns in PDAC. A, Coronal view of the mesenteric root encased by tumor cells. B, Tumor infiltrates the arterial adventitia. C, Tumor invades the arterial media but does not break through membrane elastica interna. D, Tumor invades the whole arterial wall. CHA indicates common hepatic artery; PDAC, pancreatic ductal adenocarcinoma; SMA, superior mesenteric artery; SMV, superior mesenteric vein.

We believe that the following classification can help assess the best strategy for achieving an R0 resection because the type of infiltration can dictate the need for genuine resection or sole periarterial divestment. Type 1: cancer cells infiltrate the arterial adventitia but do not break through membrane elastica externa (Fig. 3B); Type 2: cancer cells penetrate the membrane elastica externa and invade the arterial media, but do not breach the membrane elastica interna (Fig. 3C); Type 3: cancer cells invade the arterial intima and reach the arterial lumen (Fig. 3D). The first pattern of infiltration would, in theory, be treatable with periarterial divestment, whereas the remaining 3 types would require genuine artery resection.

## DISCUSSION

Our study analyzed the perioperative and oncologic outcomes of BR/LA PDAC patients with visceral vascular infiltration after pancreatic resection to achieve R0 curative resection. This work suggested that a radical approach for PDAC patients with arterial infiltration did not notably increase the R0 resection rate, and tended to result in a rather diminished prognosis in comparison to VR. The morbidity and mortality related to this radical approach were provisionally unacceptable even in an HVC where the surgeries for PDAC were performed by the experienced pancreatic surgeons.

PDAC is commonly diagnosed as a LA disease with local invasion or distant metastasis, and its 5-year survival is only 20%–27% even after receiving neoadjuvant chemotherapy followed by radical surgery.^8,37–42^ In our study, PDAC patients with AR had longer operation time, which might be due to the complexity of AR and the time-consuming intraoperative hemostasis. The longer operation time undoubtedly brought more serious surgical trauma to the patient. Although the overall complication rates did not differ between AR and VR cohorts, the bleeding severity in PDAC patients with AR was significantly greater than that in PDAC patients with VR. The higher probability of surgery-related bleeding can be caused by the technical difficulty resulting from arterial infiltration, especially when the medial and posterior margins of the pancreatic head are involved.^43^ The massive bleeding and blood transfusion would increase the incidence of intro-/ postoperative disseminated intravascular coagulation and transfusion complications. Moreover, in our study, AR was associated with hepatic complications (hepatic abscess, hepatic encephalitis, thrombosis, and sepsis) at rates as high as 17.4% (4/23). These complications undoubtedly reduced the quality of life and increased psychological trauma in patients undergoing pancreatic surgery for PDAC. In these regards alone, AR can inevitably be perilous, even in high-volume centers for pancreatic surgery.

Other centers reported that combining AR is feasible and safe in PDAC patients for attaining a longer recurrence-free and overall survival, and the postoperative morbidity and mortality did not significantly increase compared with palliative surgery.^44–46^ Similar to our findings, these studies reported a longer operation time and higher bleeding amount due to AR when compared with standard surgery without vascular resection.^44–46^ In our cohort, 4 out of 19 PDAC patients with AR died due to complications, and 9 died in follow-up with a median survival of 14.8 months. The remaining 6 still alive PDAC patients, respectively had a survival of 65, 49, 12, 13, 15, and 6 months (last due to short observation time, operation in 2020 and 2021). Heretofore, the published results on the resection and reconstruction of major peripancreatic arteries have been conflicting.^44^ In our study, in the final pathological findings, a true arterial tumor infiltration was found in only 6 out of the 19 patients with AR. As such, surgeons might tend to overestimate arterial infiltration in PDAC, which justifies the performance of periarterial divestment in selected cases.

The main limitation of our study is its low sample size of AR, and the ultimate benefit status of AR should be constantly scrutinized and updated. Considering the prognostic relevance of PDAC, R0 resection involving AR in PDAC is still of importance for improving overall survival. AR in pancreatectomy increases the feasibility of R0 resection, which remains the only option to improve long-term survival^47^ and is identified as an independent prognostic factor^.48^ Therefore, we need to better understand the extent to which surgeons should and can resect or, at least, divest arteries during PDAC surgery for attaining the best balance between radicality and prognostic benefit, while avoiding extensive traumas.

## CONCLUSIONS

The hazards coupled to AR in pancreatic surgery is an unavoidable topic even in HVCs, although AR can evolve as a key surgical approach in the future, just like VR evolved over the past decade. The poor perioperative and oncologic outcomes of BR/LA PDAC patients with AR lead to the dismal current situation. However, with an appropriate learning curve, AR can be offered to selected patients for R0 resection with acceptable postoperative outcomes.
